# Comparisons of methods for linkage analysis and haplotype reconstruction using extended pedigree data

**DOI:** 10.1186/1471-2156-6-S1-S76

**Published:** 2005-12-30

**Authors:** Shili Lin, Jie Ding, Crystal Dong, Zhenqiu Liu, Zhenxu J Ma, Shuyan Wan, Yan Xu

**Affiliations:** 1 Department of Statistics, The Ohio State University, 1958 Neil Avenue, Columbus, OH 43210, USA; 2 Battelle Memorial Institute, 505 King Avenue, Columbus, OH 43215, USA

## Abstract

We compare and contrast the performance of SIMPLE, a Monte Carlo based software, with that of several other methods for linkage and haplotype analyses, focusing on the simulated data from the New York City population. First, a whole-genome scan study based on the microsatellite markers was performed using GENEHUNTER. Because GENEHUNTER had to drop individuals for many of the pedigrees, we performed a follow-up study focusing on several regions of interest using SIMPLE, which can handle all pedigrees in their entirety. Second, 3 haplotyping programs, including that in SIMPLE, were used to reconstruct haplotypic configurations in pedigrees. SIMPLE emerges clearly as a preferred tool, as it can handle large pedigrees and produces haplotypic configurations without double recombinant haplotypes. For this study, we had knowledge of the simulating models at the time we performed the analysis.

## Background

Whole-genome scan (WGS) with microsatellite markers is currently one of the frequently used strategies in preliminary linkage analysis. For complex traits such as Kofendrerd Personality Disorder (KPD), nonparametric linkage (NPL) methods based on allele sharing statistics are popular approaches, as the underlying genetic models are complex and hard to estimate. In particular, the S-pairs statistic implemented in GENEHUNTER (GH) [1] is frequently used. However, the hidden Markov model (HMM) algorithm implemented in GH scales exponentially with the number of individuals in a pedigree, thus some individuals in larger pedigrees need to be dropped before an analysis. Skrivanek et al. [2] showed that this practice of dropping individuals may result in trimming out too much information, leading to a substantial loss of power. Thus, in the current study, we intend to evaluate whether much power is lost for an analysis using GH in this particular application. This is accomplished by analyzing all pedigrees in their entirety using SIMPLE . SIMPLE is a software package based on a Monte Carlo sequential imputation method. In particular, the S-pairs statistic has been implemented and well tested [2].

In post-genome research, as high-throughput data become increasingly available at a relatively low cost, greater attention has been given to haplotype analysis, including estimation of population haplotype frequencies and reconstruction of haplotypic configurations (HCs) in pedigrees. Haplotypes reconstructed for individuals in pedigrees can be used for association studies. They can also point to (genotyping) errors if multiple (double or more) recombinations occur in a small chromosomal segment. In the current study, we focus on comparing 2 types of methods for reconstructing haplotypes for pedigrees. One is rule/combinatorial-based; we use the PEDPHASE program [3] with the objective of finding a minimum recombinant haplotypic configuration (MRHC). The other type of method to be evaluated is probability/likelihood-based. The haplotyping routines in both GH and SIMPLE fall into this category. In GH, the HC that maximizes the posterior distribution conditional on the observed marker genotypes is inferred. In SIMPLE, the entire posterior distribution is estimated; users can obtain as many HCs (given in descending order of their estimated probabilities) as they desire. More importantly, the chi-square interference model ([4] and references therein) has been incorporated to discourage multiple recombinant haplotypes.

## Methods

### Data selection

Since New York City is the only population among the 4 in the simulated data consisting of extended pedigrees, we focused on that population. The phenotype of interest is the affection status for KPD. We performed the linkage analysis using the microsatellite markers. For the haplotype analysis, we focused on the region of interest on chromosome 3 (at the end of the chromosome) identified from our linkage analysis. Specifically, we used the last 8 3-cM-density single-nucleotide polymorphism (SNPs) on chromosome 3.

### A Monte Carlo based method

SIMPLE [2,5] is a Monte Carlo method based on sequential imputation. This Monte Carlo method is an application of importance sampling in which we sequentially impute ordered genotypes locus by locus. For NPL analysis, we further impute inheritance vectors conditional on the imputed ordered genotypes. The resulting inheritance vectors together with the importance sampling weights are used to derive a consistent estimator of an allele sharing statistic; we use S-pairs for this application. Computationally, SIMPLE scales linearly in both the number of pedigree members and the number of marker loci, hence it can make use of all available information from all pedigree members.

For haplotype analysis, the imputed ordered genotypes and the associated importance sampling weights are used to form consistent estimates of the probabilities of HCs. Thus, this likelihood-based method can examine the whole distribution of HC, and can offer multiple HCs for further consideration, such as for usage in an association study. The class of chi-square recombination models, which has been demonstrated to fit human data adequately [4], is also incorporated. In particular, we will use the model with parameter m = 4 (m4; representing the degree/intensity of interference), as this level of interference has been found to model human data well, in addition to the no interference model (m = 0; m0).

### An exact approach

We also used the software package GENEHUNTER [1] for NPL analysis based on the S-pairs allele-sharing statistic. In contrast to SIMPLE, some individuals from the larger pedigrees were dropped for computational feasibility. We evaluated whether this would lead to a loss of power in this dataset. GH was also used for haplotype analysis. It yields a HC that has the highest posterior probability. However, since GH is based on the HMM algorithm, which assumes independent recombination events, recombination models cannot be incorporated into the analysis.

### A combinatorial haplotyping algorithm

PEDPHASE [3] is a software package for inferring haplotypes from genotypes on pedigree data. The algorithms implemented in the package are based on a combinatorial formulation. Its objective is to identify a MRHC. Unlike SIMPLE or GH, both of which assume Hardy-Weinberg and linkage equilibria, no such assumptions are made for this combinatorial-based approach.

## Results

### Linkage analysis

**Figure 1:**
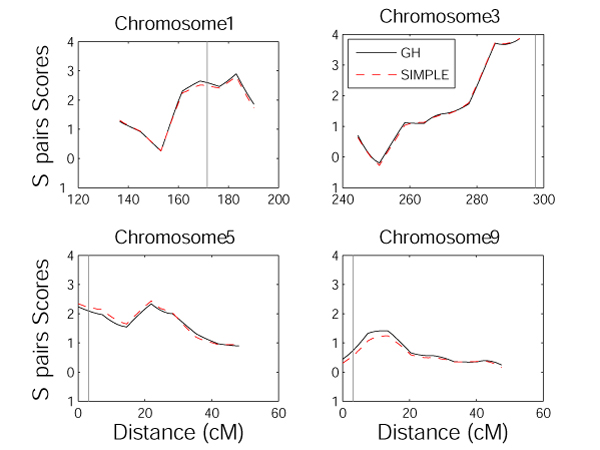
NPL score plots of GH (solid curves) and SIMPLE (dashed curves) results.

The WGS analysis using the microsatellite markers on all 4 populations and a number of replicates identified 4 regions (one of each on chromosomes 1, 3, 5, and 9) with high NPL scores from GH, although the results from New York City is the least significant. Therefore, we performed a follow-up analysis focusing on these 4 regions (based on 8 microsatellite markers in each of the regions) using the first replicate from the New York City population to see whether SIMPLE can provide any improvement, since it can use information from all individuals. Note that for this replicate, GH had to drop some individuals in 34 of the 50 pedigrees in the dataset. The results in Figure 1 show that the differences between the GH and SIMPLE score curves based on the S-pair statistic are all quite small. This is indicative of very little information contained in the additional individuals analyzed by SIMPLE. In fact, although the number of individuals dropped by GH in the 34 pedigrees goes up to 14 with a median of 4, only 2 pedigrees involve any affected individuals (1 in each) among those dropped.

### Haplotype analysis

Eight SNPs on chromosome 3, C03R0274-C03R0281, from the first New York City replicate were used in the haplotype analysis. Except for pedigrees 34 and 36, the best HCs in the other pedigrees inferred from SIMPLE (with both m0 and m4), GH, and PEDPHASE all have only single recombinations. Furthermore, the numbers of recombinations in the inferred HCs from SIMPLE match those from PEDPHASE as well as those from GH for the pedigrees that can be analyzed as a whole. The numbers of individuals dropped by GH in the other pedigrees are shown in Table 1, along with the common number of (single) recombinations for each of the 48 HCs inferred from SIMPLE and PEDPHASE.

**Figure 2:**
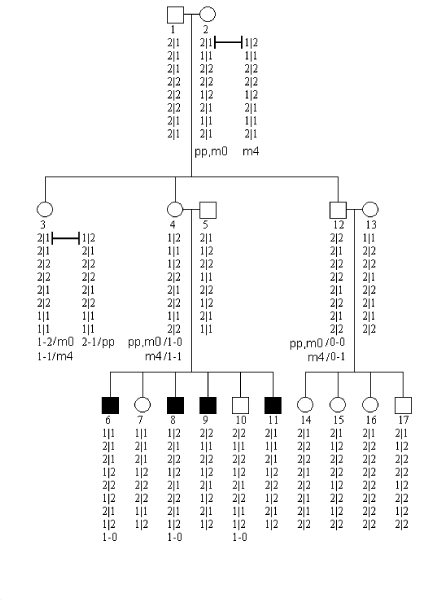
**HCs inferred from SIMPLE (m0 and m4) and PEDPHASE (pp) for Pedigree 36**. The number below each haplotype (if it exists) denotes the number of recombinations occurred to form that haplotype.

For pedigree 36, under PEDPHASE, GH, and SIMPLE m0, the HCs inferred all have a total of 7 recombinations, each including a double recombinant haplotype (paternal chromosome under PEDPHASE, maternal chromosome under GH and SIMPLE m0) in individual 3. These results are shown in Figure 2, in which the 2 linked haplotype pairs for individuals 2 and 3 show the haplotypes in each HC constructed using SIMPLE (m0 or m4) or PEDPHASE (pp). (The result from GH is identical to that from SIMPLE m0 and is thus not included in the figure.) The single haplotype pair for the other individuals is the common haplotype in all HCs from different programs/settings, although their recombination counts may be different. The occurrence of the double recombination in individual 3 could be due to the fact that such unlikely events are not penalized in the likelihood calculation under the assumption of no interference (m0). It turns out that this is indeed the case, because the double recombination vanishes under the m4 setting. The resulting HC under SIMPLE m4 has 8 recombinations; although one more than the total under SIMPLE m0 or PEDPHASE, they are all single ones.

Since GH cannot analyze pedigree 34 as a whole, we focus on the results from SIMPLE and PEDPHASE only. For SIMPLE, both m parameters lead to a HC with 7 single recombinations, although the 2 HCs differ in several individuals in the first and second generations. On the other hand, although the HC inferred by PEDPHASE also has a total of 7 recombinations, it includes a double recombinant haplotype (maternal chromosome) in individual 10, as opposed to 2 single recombinations, one on each chromosome, under SIMPLE. This shows that using MRHC as a criterion can be problematic because it may lead to a configuration with multiple recombinations within a small segment of the chromosome, a situation often regarded as potential error.

## Discussion

Although substantial power gains over GH can be obtained using SIMPLE, the differences between the SIMPLE and GH scores are small for the first replicate from the New York City population. The most likely explanation is that because there are only a couple of affected individuals among those dropped, the amount of information lost using GH is negligible.

Reconstructing HCs on pedigrees by minimizing the total number of recombinations (PEDPHASE) or maximizing the probability of the configuration (SIMPLE and GH) are 2 competing objectives. Through the analyses of the 50 New York City pedigrees, it is clear that observing the latter objective can lead to results that are comparable or better than those based on achieving the former objective (as it is currently implemented in PEDPHASE). More importantly, the ability to account for interference is a valuable asset that cannot be discounted. As we have seen here, if interference is known to exist but is being ignored, the HCs inferred (e.g., with a double recombinant haplotype) can be misleading, which may diminish the usefulness of these haplotypes in genetic studies as they may be regarded as errors.

On a different note, different HCs caused by the different formations of a founder's haplotype pair, but with similar recombination features for the rest of the individuals in the pedigree, as we see in pedigree 34, can emerge as competing configurations. Under the linkage equilibrium assumption, made by both SIMPLE and GH, these configurations may be equally probable. For markers that are closely linked, especially for SNPs within a gene, the assumption of linkage equilibrium is almost certainly to be violated. One way of eliminating the linkage equilibrium assumption in SIMPLE is to make use of population haplotype frequencies. To this end, we have investigated the performances of several programs, including SNPEM and PHASE, with a purchased package of SNPs located at the end of chromosome 3. We found that the results from all programs show strong linkage disequilibrium in the region, indicating that the linkage equilibrium assumption needs to be lifted before SIMPLE can be applied to correctly infer haplotypes based on these SNP data. However, the estimated population haplotype frequencies from different programs are inconsistent, signaling the need for further investigations before these estimated population frequencies can be incorporated into SIMPLE.

## Abbreviations

GH: GENEHUNTER

HCs: Haplotypic configurations

HMM: Hidden Markov model

KPD: Kofendrerd Personality Disorder

MRHC: Minimum recombinant haplotypic configuration

NPL: Nonparametric linkage

SNP: Single-nucleotide polymorphism

WGS: Whole-genome scan

## Authors' contributions

SL designed the study and wrote the manuscript. All authors contributed to the analyses performed, read and approved the final manuscript.

## Figures and Tables

**Table 1:** Total number of recombinations (all singles, in the recombinations column)

Pedigree	Recombinations	Drop
1	1	1
2	8	6
3	6	7
4	3	-
5	6	8
6	1	-
7	8	7
8	1	1
9	1	-
10	3	-
11	2	-
12	3	-
13	2	-
14	5	-
15	5	-
16	6	-
17	1	-
18	1	-
19	0	-
20	0	-
21	1	-
22	4	3
23	4	3
24	2	-
25	4	-
26	1	5
27	3	-
28	2	-
29	0	-
30	3	-
31	2	-
32	4	3
33	1	6
35	2	8
37	5	-
38	4	-
39	3	-
40	0	-
41	3	-
42	2	1
43	0	-
44	4	-
45	5	-
46	5	-
47	3	-
48	3	2
49	2	-
50	2	-

For the pedigrees that GH cannot analyze as a whole, the number of individuals dropped in each is also indicated (in "Drop" columns). A "-" indicates that the pedigree can be analyzed in its entirety.
